# Semantically Adaptive JND Modeling with Object-Wise Feature Characterization, Context Inhibition and Cross-Object Interaction

**DOI:** 10.3390/s23063149

**Published:** 2023-03-15

**Authors:** Xia Wang, Haibing Yin, Yu Lu, Shiling Zhao, Yong Chen

**Affiliations:** 1 School of Communication Engineering, Hangzhou Dianzi University, No. 2 Street, Xiasha, Hangzhou 310018, China; 2 Lishui Institute of Hangzhou Dianzi University, Nanmingshan Street, Liandu, Lishui 323000, China; 3 Hangzhou Arcvideo Technology Co., Ltd., No. 3 Xidoumen Road, Xihu, Hangzhou 310012, China

**Keywords:** just noticeable difference (JND), visual attention, semantic visual features, biased competition

## Abstract

Performance bottlenecks in the optimization of JND modeling based on low-level manual visual feature metrics have emerged. High-level semantics bear a considerable impact on perceptual attention and subjective video quality, yet most existing JND models do not adequately account for this impact. This indicates that there is still much room and potential for performance optimization in semantic feature-based JND models. To address this status quo, this paper investigates the response of visual attention induced by heterogeneous semantic features with an eye on three aspects, i.e., object, context, and cross-object, to further improve the efficiency of JND models. On the object side, this paper first focuses on the main semantic features that affect visual attention, including semantic sensitivity, objective area and shape, and central bias. Following that, the coupling role of heterogeneous visual features with HVS perceptual properties are analyzed and quantified. Second, based on the reciprocity of objects and contexts, the contextual complexity is measured to gauge the inhibitory effect of contexts on visual attention. Third, cross-object interactions are dissected using the principle of bias competition, and a semantic attention model is constructed in conjunction with a model of attentional competition. Finally, to build an improved transform domain JND model, a weighting factor is used by fusing the semantic attention model with the basic spatial attention model. Extensive simulation results validate that the proposed JND profile is highly consistent with HVS and highly competitive among state-of-the-art models.

## 1. Introduction

Regarding the human visual system (HVS) as a communication system with limited bandwidth and processing capacity, it is continuously receiving data input. As a result, the HVS can only sense variations in signal strength above a certain threshold, which is termed as JND [1]. Research on JND can be traced back to the experimental psychology of Ernst Weber [2] in the 19th century and was transferred to the field of digital multimedia at the end of the 20th century. Visual JND can build computational models by virtue of relevant physiology, psychology and neural research, combined with feature detection. Over the past decades, JND has proven to be a multi-factorial problem, including contrast sensitivity function (CSF), luminance adaptation (LA), masking effects and visual attention. An overview of these factors can be found in the survey by researchers in [3]. Using the computational domain as a classification criterion, there are two branches of existing JND models, i.e., the pixel domain (where JND thresholds are computed directly for each pixel) [4,5,6,7,8,9,10,11,12,13] and the transform domain (where the image is first transformed into a subband domain, and then JND thresholds are calculated for each subband) [14,15,16,17,18,19,20,21,22,23,24]. Nevertheless, both model types generally follow the same design philosophy of simulating the visual-masking effects of several elements before combining (multiplying or adding) them to obtain an overall JND estimation. The pixel-domain JND model has undergone a protracted evolution. Based on the study of luminance and contrast, the pioneering work of Chou et al. [4] computed luminance adaptation and contrast masking (CM), using the winner as the JND thresholds. Yang et al. [5] considered the overlap effect between LA and CM, and proposed a nonlinear additivity model of masking (NAMM). Furthermore, the image can be decomposed into plain, texture and edge regions for more accurate modeling of CM [6]. Since the visual sensitivity decreases with increasing retinal eccentricity, the fovea masking was introduced [7]. Inspired by the internal generation mechanism, the masking effects of ordered and disordered components were quantified based on free energy theory [8], and the pattern masking was extended sequentially [9]. In addition, the RMS contrast was used as the spatial CSF of JND in the pixel domain [10]. Based on hierarchical predictive coding theory, self-information and information entropy were utilized to calculate the perceptual surprise and perceptual suppression effects at different levels to improve the accuracy of the JND model [12].

Transform coding is a widespread means of mainstream image/video coding, and transform domain JND modeling is also a significant research area. In 1992, Ahumada [14] proposed the first DCT domain JND model combining spatial CSF and LA. Based on [14], Watson [15] introduced the CM effect and proposed the DCTune model, which laid the research foundation for the DCT domain JND modeling. Subsequently, a more realistic LA function was proposed to improve JND estimation by integrating a block classification (plain, edge, and texture) strategy [16]. For video, corresponding video JND models for the DCT domain were proposed considering spatiotemporal CSF and eye movement compensation [17], LA effect based on gamma correction and CM effect based on more accurate block classification [18], texture complexity and frequency, visual sensitivity and visual attention [19], the various sizes of DCT blocks (from 4 × 4 to 32 × 32) [20], motion direction [21], fovea masking [22], temporal duration and residual fluctuations [23].

In recent years, machine-/deep-learning-based modeling of visual perception has become a new research trend due to the rapid development of deep neural networks [25,26,27,28,29,30,31,32,33]. To fill the vacancy of JND databases for image and video compression, several scholars have suggested MCL-JCI [34], JND-pano [35], MCL-JCV [36], VideoSet [37], etc. Based on these datasets, various modeling techniques for JND eventually emerged, most notably subjective data regression [25,27], binary classification [26], picture/video-wise JND (PWJND/VWJND) or satisfied user ratio (SUR) modeling [27,28,29,30], and finding appropriate weighting factors for the JND models [25,26]. In particular, motivated by the smoothing performance (non-smoothed regions have better capability for hiding noise than smoothed regions), Wu et al. [31] proposed an unsupervised learning method for generating JND images in the pixel domain. Considering that JND should be evaluated in the human brain perception domain, Jin et al. [33] presented an HVS-based signal degradation network and employed visual attention loss to further regulate JND estimation. In addition, JND modeling also extends to machine vision [38,39]; however, this specific topic is outside the scope of this paper. Visual attention is a key attribute of HVS [40], and models combined with visual attention can improve the accuracy of JND threshold. In a study by Chen et al. [7], attention-induced fixation was detected and a scheme was designed to reflect the increase in JND with increasing retinal eccentricity. Video JND estimation for smooth pursuit eye movements (SPEM) and non-SPEM situations was accomplished by investigating the relationship between JND thresholds and parameters (spatial frequency, eccentricity, retinal motion velocity) [22]. Considering the special focus of HVS on people, Wang et al. [41] combined Itti’s [42] and Judd’s [43] attention model (with face detector and person detector) to adjust the JND threshold.

The main feature of the HVS attention mechanism is the information-selection process, in which only a small number of visual signals are transmitted to the brain for processing. Indeed, the object-based attention theory suggests that humans are drawn to objects and advanced concepts [44]. Although Wang at al. [41] considered the advanced semantics of faces, there are often more advanced semantics in images than in plain faces. In any case, traditional visual attention models mainly consider low-level features such as color, luminance, and orientation, while underestimating high-level semantic information, which leads to reduced JND model accuracy under the attention mechanism of HVS. This is mainly due to the difficulty of semantic feature extraction and fusion. Meanwhile, it is well known that deep neural networks are full of semantic information [45], but current JND models based on deep learning mainly try to construct PWJND/VWJND or SUR without fully considering semantic character behaviors.

In addition, neurons transmit and express visual information by consuming energy [46]. The structure and function of neural networks follow the essential principles of resource allocation and minimization of energy constraints. Therefore, not all stimuli trigger neuronal responses, i.e., biased competition for visual attention influences neuronal activity [47]. The theory of biased competition suggests that selectivity of one (or more) points in the visual process is caused by interaction between visual objects for neural representations. More interestingly, with the limited attentional resources, there will always be certain items and their visual features that win out in the competition. Therefore, it is inevitable to consider such interaction while constructing a semantics-based attention model.

From the above discussion, it is clear that semantics is crucial for accurate estimation of JND thresholds. Furthermore, it should be noted that this paper concentrates on the tuning scheme for DCT-based JND estimation because the majority of image/video processing, especially in image/video compression, is conducted in the DCT domain. Particularly, this paper argues that there are three semantic features to be considered in the attention allocation problem of videos, namely, object, context, and cross-object. Since it is troublesome to extract semantic attributes manually, this paper uses grad-CAM [48] and VNext [49] to extract semantic objects and features through deep learning. Based on this, attention effects induced by object-based semantic features are analyzed, including the sensitivity and size of semantic objects and central bias. Second, contextual information is another vital element that enhances instance differentiation and is considered as a form of center-surround contrast. Furthermore, considering limited attentional resources and infinite information, this paper describes the attentional competition effects of different semantic features (including focus intensity, spatial distance, and relative area) in terms of cross-objects to achieve an accurate model of semantic attention.

Specifically, inspired by the framework of [23], this paper proposes a statistical probability model corresponding to the feature parameters and quantifies the response of visual attention in a perceptual sense using information theory. Then, the attentional competition factor for calibrating the semantic attention model is proposed. Finally, the adaptive semantic attention weight is obtained by unifying and fusing the perceptual feature parameters, and the JND model is adjusted using this weight.

The rest of this paper is organized as follows. Section 2 presents three features and describes the quantification strategies for these parameters. Section 3 analyzes the interaction between the objects and context. Section 4 depicts the fusion approaches of these parameters together with the competition properties of attention. Section 5 details the proposed JND profile. Simulation results and conclusions are given in the Section 6 and Section 7, respectively.

## 2. Object-Wise Semantics Parameter Extraction and Quantification

As stated, the literature lacks a clear understanding of the mechanism of interaction between semantic perception and semantic features. This motivates us to formulate an effectual JND profile leveraging the HVS semantic traits, which is subject to challenges. The first issue is how to precisely extract and quantify the feature parameters that thoroughly depict the perceptual response of semantic HVS features in videos, such as semantic sensitivity, objective area, central bias.

### 2.1. Semantic Instances Extraction

**Figure 1:**
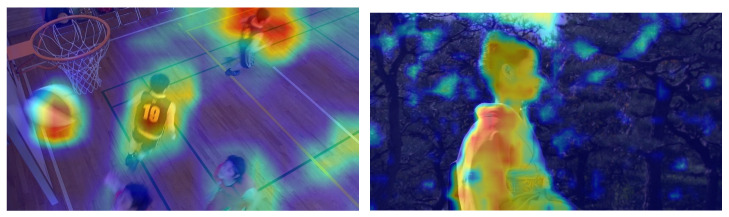
The grad-CAM heat-map results.

**Figure 2:**
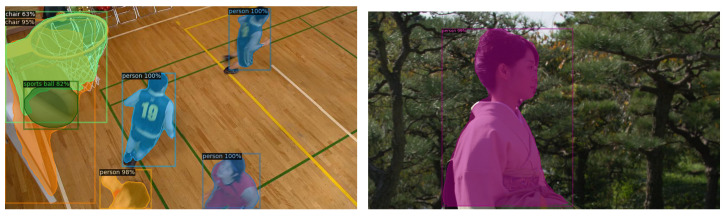
The VNext extraction results.

Accurate extraction of high-level semantic features of videos helps to better visual attention modeling. On that basis, this paper uses grad-CAM for the extraction of high-level semantic features. In this network, the further the layer, the ampler the semantics is and the more spatial information can be preserved. In this respect, grad-CAM contains all the semantics of our target of interest. For more details, please see [48]. With grad-CAM, we can draw heat maps which are shown in Figure 1 below. Moreover, to effectively utilize the various properties of semantic objects, this paper uses VNext [49] to extract semantic objects, and the results are shown in Figure 2.

### 2.2. Semantic Sensitivity Quantification

The research [50] has shown that attention is focused on informative content. One definition of attention describes it as a limited resource for information processing, with the area of interest carrying the bulk of the information transmitted to the brain. Hence, Bruce et al. [51] pointed out that Shannon self-information can be used to outline attention. Whereupon, the attention value of a pixel located at (x,y) in a video frame is defined below.

(1) $$A\left(x,y\right)=-\log_{2}P\left(F=f\left(x,y\right)\right)$$

where *F* denotes a random variable of the feature of pixel (x,y), f(x,y) represents the image features of pixel (x,y), P(F=f(x,y)) indicates the probability of feature *F*.

The concept of constructing statistically sparse representations of images appears to be fundamental to the primate visual system, as evidenced by a large body of research. That is, to some extent, computing the probability distribution of features is essentially a matter of statistical feature sparsity. If a pixel associated with a feature has a lower probability of appearing, it has a better chance of attracting attention because it conveys a greater amount of information. In view of this, the probability density function (PDF) of feature *F* is measured as follows by performing histogram statistics on the whole picture.

(2) PDF=hist(F=f(x,y))=P(F=f(x,y))

Accordingly, the initial attention based on semantic sensitivity (*m*) can be calculated as follows.

(3) $$I\left(m\right)=-\log_{2}hist\left(f\left(m\right)\right)$$

**Figure 3:**
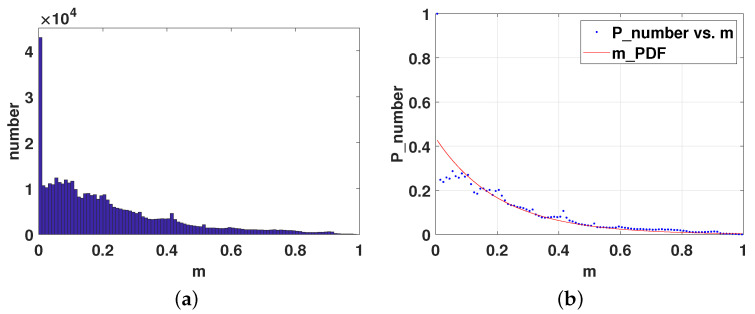
(**a**) A semantic sensitivity histogram; (**b**) A semantic sensitivity PDF curve.

It is worth noting that instead of using a uniform-fitting function, to better suit the image content, the histogram of each frame is adaptively fitted. Figure 3 shows the histogram of the 2nd frame in the HEVC standard test sequence “BasketballDrill” and its PDF fitting curve, separately.

### 2.3. Objective Area Quantification

**Figure 4:**
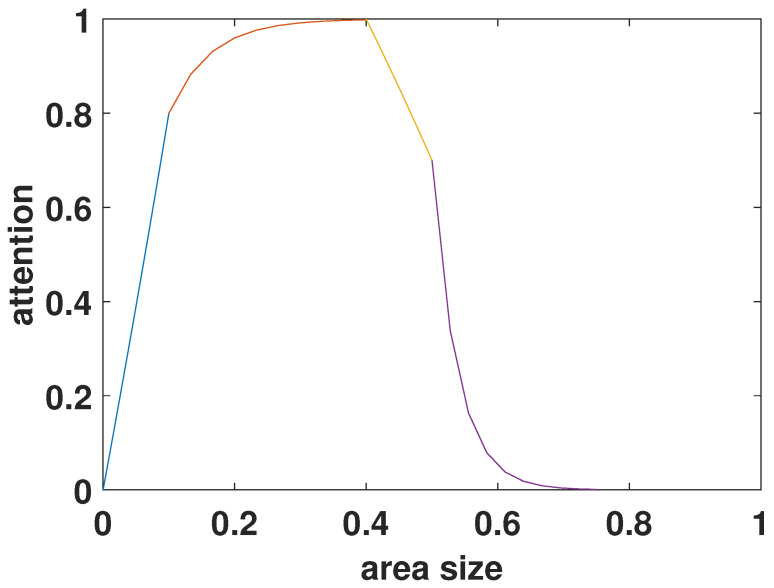
The function map of Equation (4).

The literature demonstrates that the larger the area, the higher the probability of attention, i.e., the probability of attention of semantic objects is proportional to size, however, this property becomes smooth after a certain saturation threshold [52]. This relationship can be summarized below using a piecewise function [53], with values within [0, 1].

(4) $$-\log_{2}\left(p\left(\eta_{j}\right)\right)=\left\{\begin{array}{c} -\frac{\ln(1-\eta_{j})}{c_{1}}\;0\leq \eta_{j}\leq t_{1}\\ 1-\exp\left(-c_{2}\cdot \eta_{j}\right)\;t_{1}<\eta_{j}\leq t_{2}\\ s_{2}+c_{3}\ln\left(1-\eta_{j}+t_{2}\right)\;t_{2}<\eta_{j}\leq t_{3}\\ s_{3}\cdot \exp\left(-c_{4}\left(\eta_{j}-t_{3}\right)\right)\;t_{3}<\eta_{j}\leq t_{4}\\ 0\;t_{4}<\eta_{j}\leq 1.0 \end{array}\right.$$

where 0<j≤N, *N* is the number of semantic objects in an image frame, $\eta_{j}$ represents the size of semantic object, which is calculated as: $\eta_{j}=B_{j}/\left(W\times H\right)$, $B_{j}$ represents the number of objective pixels, and W,H refer to the width and height of the image frame. Figure 4 shows the relationship between area and attention.

Likewise, for the same size semantic objects, attention will be affected by the aspect ratio. As is known to all, the aspect ratio of current popular displays is 16:9. It follows that the closer the aspect ratio of a semantic object is to our vision field, the more it attracts attention.

**Figure 5:**
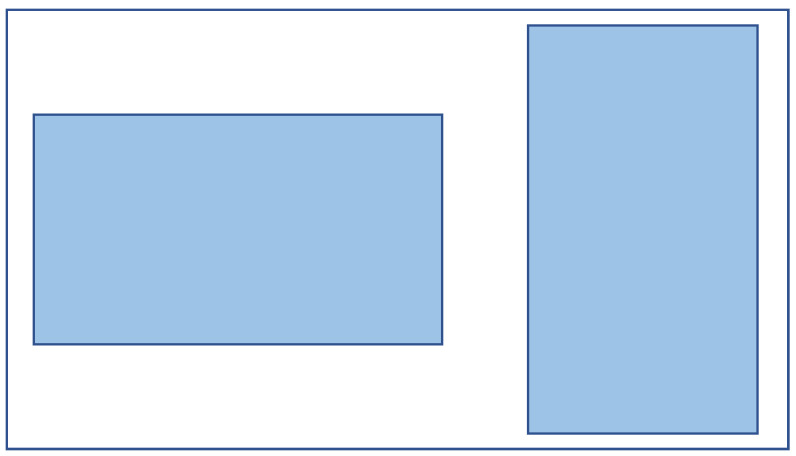
The illustration for the aspect ratio.

Figure 5 is an illustration for the aspect ratio. For a more clear description, the blue rectangle on the left is labeled as A, the one on the right is B, and the container containing these two rectangles is C. Despite having the same area, A and B have various aspect ratios. The aspect ratios of A and C are 16:9, while B is 9:16. It is obvious from the example that rectangle A attracts more attention than rectangle B. Note that the rectangle is used merely for convenience of observation, and the results hold true other shapes as well.

Therefore, the joint effect of semantic object size, the aspect ratio ($R_{r}$) is used to measure the attention of semantic object size.

(5) $$I\left(\eta_{j}\right)=-\log_{2}\left[p\left(\eta_{j}\right)\right]-\alpha_{1}\log_{2}{\left(\frac{R_{r_{j}}}{\beta_{1}}\right)}^{\beta_{2}}$$

where $\alpha_{1}$, $\beta_{1}$, $\beta_{2}$ are all adjustment control parameters, $r_{j}$ denotes the aspect ratio of the *j*-th semantic object and is normalized to [0, 1].

The model is consistent with HVS subjective perception that HVS perceptual attention intensity increases with the size of the object but there is a saturation point. The reason is that when the object area is too large, the target and background are non-separable, i.e., the target is likely to be the background component at this point. In addition, targets with an aspect ratio closer to 16:9 attract greater HVS attention, which raises perceptual sensitivity and lowers JND thresholds.

### 2.4. Central Bias Quantification

It is stated that most images have the foreground object in the center of the image frame [43]. It follows that when a person looks at an image, he or she habitually looks at the center of the image first. Eye-movement experiments have shown that HVS tends to focus on the center of the image, and the deviation from the target center to the image center, i.e., the central bias, has been considered a noteworthy prior in attention modeling [44]. Based on this prior knowledge, many attention modeling algorithms based on central bias use various methods to increase the attention value at the image center location as a way to highlight the salient targets in the image.

Nevertheless, the feedback brought by the central bias is not always favorable. For some unconventional cases where the target is off-center in the image, attention enhancement at the image center can lead to over/under estimation of the target far from the image center. In general, the problem with the classical center-priority based attention modeling algorithm is that it tends to lack flexibility, which leads to significant inaccuracies when the foreground targets of the image are not positioned as expected.

The formation of a top-notch attention map is the ultimate goal of attention modeling. The complexity of attention modeling is greatly reduced and the quality of the final attention map is improved if the locations of the salient targets are approximated before proceeding to formal attention modeling.

In this letter, the object (*M*) closest to the image center is calculated, and the central bias is constructed with *M* as the experienced center of vision. The closer an object is to the visual center, the higher probability it is to be noticed, i.e., the attention probability of semantic objects is inversely proportional to the central bias distance, which is denoted as follows.

(6) $$P\left(A|d_{j}\right)=1-\log2\left[1+{\left(\frac{d_{j}}{\omega_{1}}\right)}^{\omega_{2}}\right]$$

where $d_{j}$ is the distance from the semantic object to *M*, and normalized to [0, 1].

(7) $$d_{j}=mean\left(\sum_{f\in O_{j}}^{}{∥\left(x_{f},y_{f}\right)-\left(x_{M},y_{M}\right)∥}^{2}\right)$$

here, $O_{j}$ is a semantic object, *f* denotes the pixel of $O_{j}$, $\left(x_{f},y_{f}\right)$ represents the coordinates corresponding to *f*, $\left(x_{M},y_{M}\right)$ means the center coordinates of *M*, $∥\cdot ∥$ is the euclidean distance, $\omega_{1}$, $\omega_{2}$ serve as the adjustment parameters.

**Figure 6:**
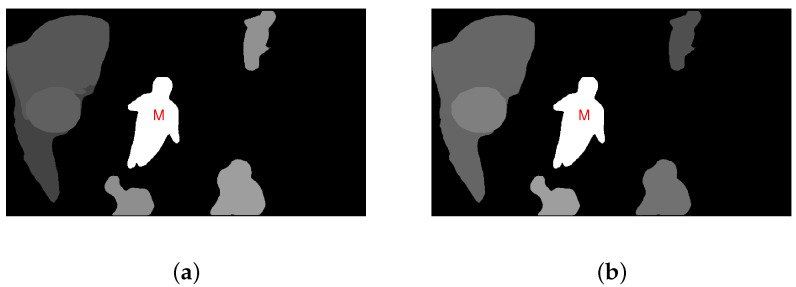
(**a**) The traditional central bias map; (**b**) The modified central bias map.

As illustrated in Figure 6, the modified central bias result is better in line with HVS perception. Since the target *M* in the figure is closest to the image center, it obviously draws the highest attention, which is shown by the brightest brightness. In this instance, the target *M* stands in for the image center as the experienced center of vision and the closer the other objects are to the target *M*, the more attention they attract, and the brighter they are presented in the figure.

## 3. Context-Wise Attentional Inhibition Quantification

Given that HVS still beats the state-of-the-art computer vision systems, modeling visual attention in natural scenes is a hot topic of research today. How is such great effectiveness achieved? The ability of HVS to utilize the context of a scene to direct attention before most items are recognized appears to be a key factor compared to artificial systems [54].

How does the context of a scene serves to focus attention on objects in the scene? Davenport and Potter [55] suggest that there is an interaction between objects and context during scene processing, which is supported by research. On this basis, this paper conceptualizes the local spatial arrangement of the image background as an alternative to the context and focuses primarily on the complexity of the patterns.

In most situations, the region between the target and its background is heterogeneous and has a complex organization. The density of the visual information presented is normally used to describe the complexity of the visual background. In general, a scene is complex when its background displays a large amount of information and the unpredictability of this information is considerable [56]. Typically, the complexity of the visual context usually has a detrimental impact on the visual task. The higher the visual complexity, the longer the response latency of the nerve cells and the stronger the degree of inhibition of the target [57].

**Figure 7:**
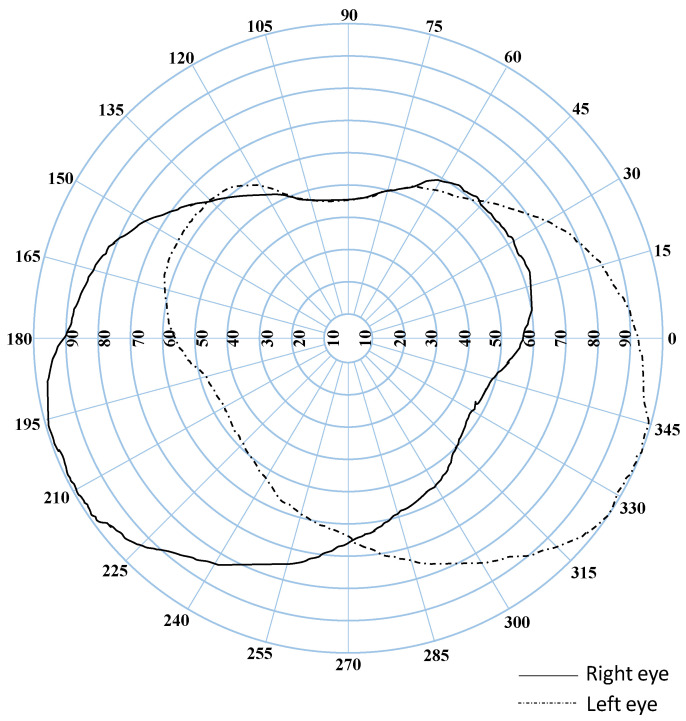
Areas of vision field.

As the contextual complexity of the target suppresses the attention strength, the more complex the pattern of the background, the stronger the suppression, while this is going on, the findings of numerous human visual field experiments indicate that the human binocular visual field is approximately circular (oval), with the proximate form depicted in Figure 7 [58]. Hence, to represent the inhibition of each semantic target background, we utilize the average of the contextual complexity in the circumcircle of semantic targets to simplify the calculation. Since entropy reflects the chaotic degree of a system, we estimate contextual complexity by local entropy. As a result, attention and contextual complexity are related in the following way.

(8) $$P\left(\bar{A}|u_{j}\right)=\log2\left[1+{\left(\frac{u_{j}}{\xi_{1}}\right)}^{\xi_{2}}\right]$$

where $u_{j}$ is the contextual complexity, and normalized to [0, 1].

(9) $$u_{j}=mean\left(entfilt\left(circle\left(O_{j}\right)-O_{j}\right)\right)$$

here, $\xi_{1}$ and $\xi_{2}$ serve as the scaling factors, circle(·) denotes circumcircle, entfilt(·) is the function to calculate the local entropy.

**Figure 8:**
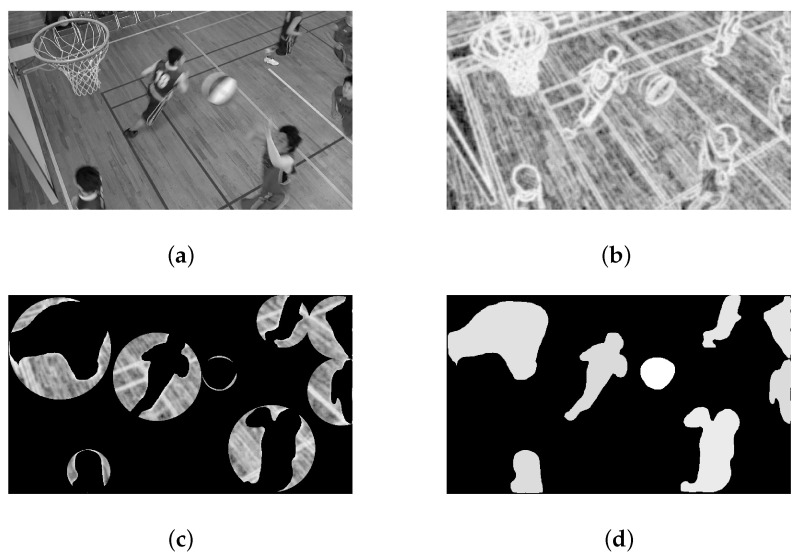
(**a**) The 212th original frame of the “Basketball Drill”; (**b**) The complexity map of the whole frame; (**c**) The contextual complexity map of the semantics; (**d**) The inhibitory effect of contextual complexity on semantics.

Figure 8 shows the inhibitory effect of contextual complexity on semantics.

## 4. Fusion Strategies and Cross-Object-Wise Attentional Competition

Furthermore, given elaborately chosen feature parameters, the second sore point is how to quantify the interaction among these feature parameters, i.e., how to fuse these heterogeneous feature parameters.

Consolidating the semantics-based attention model is the aim of this part. In general, different feature maps do not have the same attributes, and different features contain complementary global contextual information and local detailed information between them. Consequently, to obtain reliable results for the attention distribution, it is essential to select the appropriate weights for each feature map.

In this paper, to estimate the weights of the feature maps above, the gradient change value of each feature map is first calculated, where the greater the variation of the data, the more information can be gleaned. The gradient variation is then used to estimate the relative weights of these feature maps. Finally, utilizing their individual weights, the feature maps are normalized and combined into a single attention map.

(10) $$att_{pre}=\frac{\lambda_{1}\cdot I\left(m\right)+\lambda_{2}\cdot I\left(\eta \right)+\lambda_{3}\cdot P\left(A|d\right)}{P(\bar{A}|u)}$$

here, $\lambda_{i}\left(i=1,2,3\right)$ serves as the weighting factor, and is calculated as follows.

(11) $$\lambda_{i}=\frac{vg(z_{i})}{\sum_{i=1}^{3}vg(z_{i})}$$

(12) $$vg\left(z_{i}\right)=\frac{\max\left(g\left(z_{i}\right)\right)-\min\left(g\left(z_{i}\right)\right)}{mean(g\left(z_{i}\right))}$$

(13) $$g\left(z_{i}\left(O_{m}\right)\right)=\sum_{m=1,m\neq n}^{N}\left|z_{i}\left(O_{m}\right)-z_{i}\left(O_{n}\right)\right|$$

where $z_{i}\in \left\{I\left(m\right),I\left(\eta \right),P\left(A|d\right)\right\}$ is the attention map of different semantic features.

**Figure 9:**
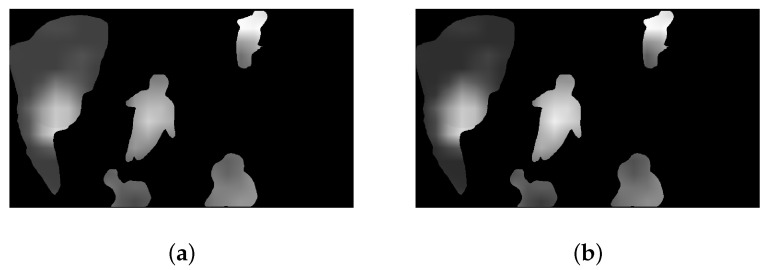
(**a**) The initial attention map with the identical weights; (**b**) The initial attention map with the gradient variation based weights.

Figure 9 displays the initial attention map with different weighting factors. As illustrated, compared to map (a), the map (b) with the gradient variation based weights is more consistent with the HVS subjective perception. This is partly due to the fact that we fully take the content characteristics of different feature maps into account.

However, there is a competitive mechanism that manifests itself as a relative enhancement of responses to task-relevant objects or a relative inhibition of neglected objects within the brain [59]. That is, when modeling the semantic attention model, it is not sufficient to consider only the semantic object itself, but also to take full account of the biased competition between different objects.

- Focused Intensity: when multiple stimuli are present in the visual field at the same time, inhibitory competition in the visual cortex affects the allocation of attention, which probably stem from the inability to bias the interaction toward a particular object [60]. In reality, the brain’s representation of information is essential for human vision, and regardless of the distance between neurons or neuronal populations, they do not operate independently, but constantly interact with each other. Because their activities are interconnected and competing, they cannot provide “independent attentional resources” [61]. That is, when there are multiple semantic objects in an image, attention to each semantic object is impacted by the other objects. The higher the intensity of the focus, the more pronounced the suppression of the other objects;
- Spatial Distance: event-related potentials, functional MRI investigations as well as single-cell recordings in monkeys [62] and humans [63] have pointed out that neural enhancement in attentional focus may be followed by neural inhibition in peripheral regions. Detection is slower and discrimination performance is poorer at interference locations close to the target compared to interference locations far from the target [64]. In the receptive fields of cells, shifting attention from one stimulus to another can have a strong effect when two stimuli are in close proximity to each other. For stimuli that are far apart, the effect is much less [59]. In other words, the more spatially distant the objects are from each other, the smaller the inhibitory impact;
- Relative Area: since an object generally has several neighbors, the relative area of an object shows with its neighbors affects its attention competition. The rationale for selecting relative area is that, even if the object has an attractive intrinsic area, it may not stand out unless it exhibits the greatest contrast if all of its neighbors also have attractive areas [53]. In particular, the higher the area contrast, the more strongly other objects will be suppressed.

Based on the points discussed above, the attentional competition weight is defined as follows:

(14) $$C_{A}\left(k\right)=\left(\sum_{k=1,k\neq l}^{N}D_{\tau }\left(k,l\right)timesD_{\nu }\left(k,l\right)\right)\cdot S_{\delta }\left(k\right)$$

in which, $D_{\tau }\left(k,l\right)$, $D_{\nu }\left(k,l\right)$ are the average focused intensity distance and the spatial proximity distance between the *k*-th, *l*-th semantic objects, respectively.

(15) $$D_{\tau }\left(k,l\right)=\exp\left(\frac{F\left(O_{k}\right)-F\left(O_{l}\right)}{F\left(O_{k}\right)+F\left(O_{l}\right)}\right)$$

(16) $$F\left(O_{k}\right)=mean\left(att_{pre}\left(O_{k}\right)\right)$$

where $F(O_{k})$ denotes the average focused intensity fo the *k*-th object.

(17) $$D_{\nu }\left(k,l\right)=\exp\left(\frac{\mu }{{∥\left(x_{k},y_{k}\right)-\left(x_{l},y_{l}\right)∥}^{2}}\right)$$

where μ is a scaling factor, $\left(x_{k},y_{k}\right)$ and $\left(x_{l},y_{l}\right)$ are the coordinates of the center point of the *k*-th, *l*-th semantic objects.

(18) $$S_{\delta }\left(k\right)=1-\exp\left(-12\cdot G\left(k\right)\right)$$

where $S_{\delta }\left(k\right)$ represents the relative area competition weight of the *k*-th semantic object, G(k) is the relative area of the *k*-th object with respect to its neighbors, see [53] for more details.

As a result, the semantics-based attention model is constructed by melting the attentional competition weight.

(19) $$Sem_{att}=att_{pre}\cdot C_{A}$$

**Figure 10:**
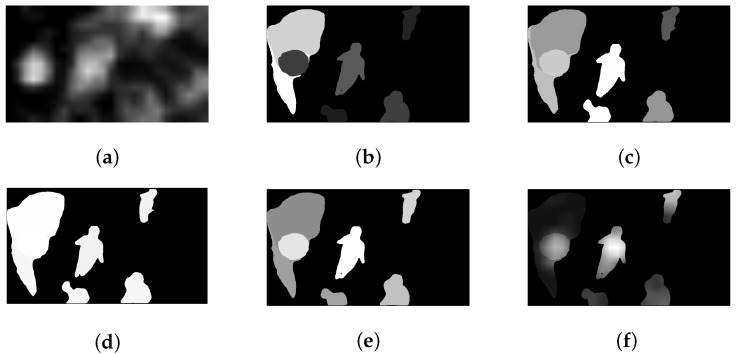
(**a**–**c**) The attention map of the semantic sensitivity, objective area, central bias; (**d**) The inhibition effect of the contextual complexity; (**e**) The attentional competition map; (**f**) The semantic attention map.

Figure 10a–c takes the 2nd frame of the ”Basketball Drill” sequence as an illustration, showing the attention map of semantic sensitivity, objective area and central bias. Figure 10d displays the inhibition effect of the contextual complexity. Figure 10e shows the attentional competition result. Figure 10f exhibits the final attention map. Intuitively, brighter areas indicate higher attention, inhibition and competition.

## 5. Semantic-Based Spatio-Temporal Transform Domain JND Profile

**Figure 11:**
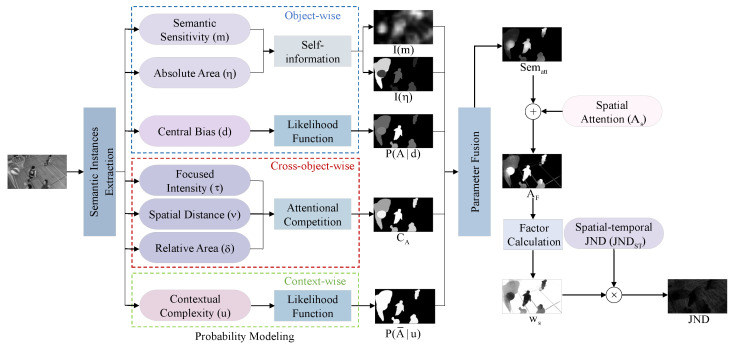
A briefoverview of the proposed spatio-temporal JND model.

As mentioned above, this paper combines semantic sensitivity, objective area, central bias, contextual complexity and attentional competition to measure semantic perceptual attention. Then, based on the model in [65], considering the impact of semantics, this study modifies the spatial attention factor, aiming for a more accurate JND model. The framework of the proposed JND profile is shown in Figure 11.

By introducing the weighting factor of the semantics-based attention, we propose the following JND model.

(20) $$JND\left(t,n,i,j\right)=JND_{st}\left(t,n,i,j\right)\cdot w_{s}\left(t,n\right)$$

here, $JND_{st}\left(t,n,i,j\right)$ is the spatio-temporal JND threshold of coefficient (i,j) of the *n*-th block in the *t*-th frame, considering the LA, CSF and masking effects [23].

Taking the spatio-temporal JND threshold $JND_{st}$ as the basis, this work proposes a patch-wise weighting factor, the attentional weight $w_{s}\left(t,n\right)$, accounting for the visual perceptual attention of semantics, aiming at developing a more accurate JND model. Patch level $w_{s}\left(t,n\right)$ is determined as the mean of pixel-wise adjustment factors in the spatial domain, $w_{s}\left(t,i,j\right)$, of the *n*-th image block in the *t*-th frame.

To be specific, by following [65], we estimate spatial attention $A_{s}\left(x\right)$, and combine semantic attention $Sem_{att}\left(x\right)$ to get semantics-based spatial attention $A_{F}\left(x\right)$ using NAMM [5].

(21) $$A_{F}\left(x\right)=A_{s}\left(x\right)+Sem_{att}\left(x\right)-0.3\cdot \min\left(A_{s}\left(x\right),Sem_{att}\left(x\right)\right)$$

In general, the larger the visual attention $A_{F}\left(x\right)$, the smaller the JND threshold. Intuitively, it seems sensible to apply the sigmoid-like function to normalize $A_{F}\left(x\right)$ to [0, 1] and measure the spatial attention weight $w_{s}\left(t,n\right)$ below.

(22) $$w_{s}\left(t,n\right)=\vartheta_{1}\cdot \left(1-\frac{1}{1+\exp(-\vartheta_{2}\cdot A_{F}\left(t,n\right))}\right)$$

where $\vartheta_{1},\vartheta_{2}$ are normal constants, $A_{F}\left(t,n\right)$ is the mean of the *n*-th block of $A_{F}\left(x\right)$. According to subjective experiments, $\vartheta_{1}$ and $\vartheta_{2}$ are set to 2.5 and 2, respectively.

**Figure 12:**
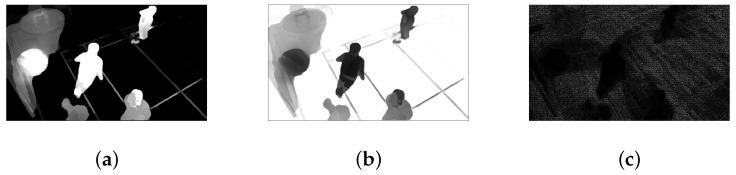
(**a**) The semantic-based spatial attention $A_{F}\left(x\right)$ map; (**b**) The spatial attention weight $w_{s}\left(x\right)$ map; (**c**) The proposed JND threshold map.

Figure 12a,b display the obtained attention map and the corresponding weight map. Figure 12c shows the final spatio-temporal JND threshold map. In Figure 12a,c, brighter areas indicate higher visual attention or masking, while the opposite is true for Figure 12b.

## 6. Experimental Results

### 6.1. Comparison of Model Performance

An ideal JND model should, in a sense, distribute noise more fairly, concealing more of it, with acceptable perceptual quality. Thus, we add coefficient-wise JND-guided noise to video sequences, as described in [22], to assess the effectiveness.

(23) $$\hat{R}\left(t,n,i,j\right)=R\left(t,n,i,j\right)+\rho \cdot rand\left(t,n,i,j\right)\cdot JND\left(t,n,i,j\right)$$

where R(t,n,i,j) is the transform coefficients of original sequence, $\hat{R}\left(t,n,i,j\right)$ is the JND noise contaminated coefficients, ρ regulates the energy of JND noise, and rand(t,n,i,j)∈{+1,−1} is a bipolar random noise.

**Figure 13:**
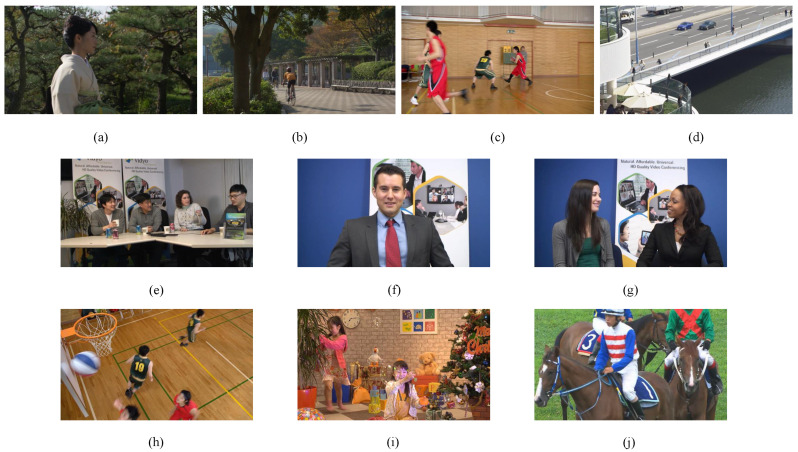
The HEVC standard test sequences. (**a**) Kimono1; (**b**) ParkScene; (**c**) Basketball Drive; (**d**) BQTerrace; (**e**) FourPeople; (**f**) Johnny; (**g**) KristenAndSara; (**h**) Basketball Drill; (**i**) PartyScene; (**j**) RaceHorses.

We evaluate the effectiveness of the proposed work in comparison to four benchmark models, namely Bae 2017 [22], Zeng 2019 [66], Wang 2020 [12], Xing 2021 [23], and Li 2022 [67]. Ten videos with diverse semantics were chosen from the HEVC standard sequences in order to meet the requirements of different resolutions. Four of them are videos with 1920 × 1080 full HD resolution, namely, “Kimono1”, “ParkScene”, “Basketball Drive” and “BQTerrace”, as shown in Figure 13a–d. Three of them, “FourPeople”, “Johnny”, “KristenAndSara”, are 1280 × 720 resolution videos, as shown in Figure 13e–g. The remaining three, “Basketball Drill”, “PartyScene”, and “RaceHorses”, are videos with 832 × 480 resolution, as shown in Figure 13h–j.

Using Peak Signal-to-Noise Ratio (PSNR) as an objective evaluation criterion, a lower PSNR indicates a better ability to mask noise. However, despite that PSNR is the most popular objective quality evaluation metric, the results of a large number of empirical-psychological studies have demonstrated that PSNR scores fall short of properly capturing HVS perception due to the involvement of visual physiological and psychological mechanisms. Furthermore, the human eye is the receiver of the final visual signal, and therefore, subjective quality metrics must be taken into account when evaluating the proposed JND model.

In this paper, the visual quality of JND-contaminated videos was assessed by recruiting 17 subjects with normal or corrected normal vision, using the subjective viewing test in [68]. Specifically, the monitor used for the display was a 27-in LED monitor, while the view distance was six times the height of the video frame. The difference between the original and processed sequences was observed using the Double Stimulus Continuous Quality Scale (DSCQS) method [23].

**Figure 14:**
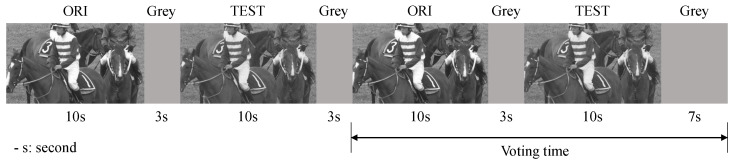
DSCQS method, where the original sequence (ORI) and test sequence (TEST) are pseudo-randomly ordered for each presentation.

Figure 14 illustrates the testing procedure of the DSCQS. In the experiment, for each presentation, the reference and test sequences are arranged in a pseudo-randomized form. During the voting period, participants are expected to rate the quality of each of the two videos. The visual quality of the JND-contaminated videos is then measured by calculating the MOS difference (DMOS) between the original and matched processing sequences. The calculation is described below.

(24) $$\mathrm{DMOS}=MOS_{JND}-MOS_{ORI}$$

where $MOS_{ORI}$ and $MOS_{JND}$ are the measured average opinion score values for the original and test videos, respectively. Five quality scales are used, i.e., excellent (80–100), good (60–80), fair (40–60), poor (20–40), and bad (0–20). The smaller the value of DMOS, the better the visual quality of the JND-polluted video.

**Figure 15:**
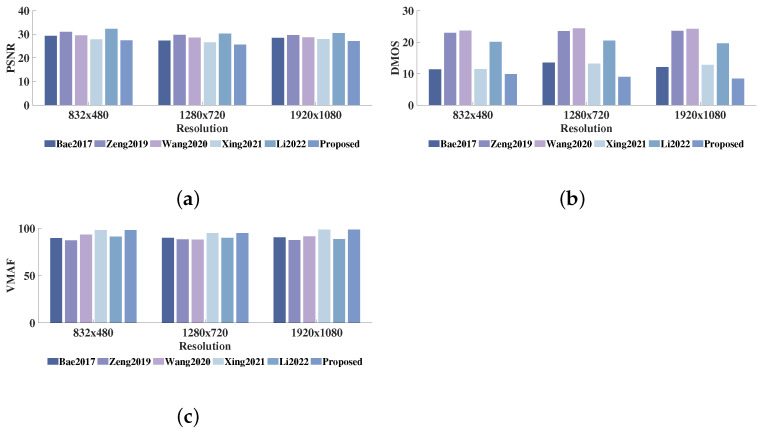
(**a**) Average PSNR values in Table 1 for 832 × 480, 1280 × 720, and 1920 × 1080; (**b**) Average DMOS values in Table 1 for 832 × 480, 1280 × 720, and 1920 × 1080; (**c**) Average VMAF scores in Table 2 for 832 × 480, 1280 × 720, and 1920 × 1080 [12,22,23,66,67].

The detailed performance results of different JND models are shown in Table 1. From the panoramic viewpoint, it can be found that the proposed JND model has the best performance on PSNR and DMOS for all videos except the “RaceHorses” sequence. This is mainly due to the large proportion of foreground semantic objects in the “RaceHorses” sequence, while our model guides only a small amount of noise into the foreground semantic object region. Therefore, the PSNR value of the proposed JND model is slightly higher than this of Xing, 2021. In addition, as shown in Table 2, the Video Multimethod Assessment Fusion (VMAF) scores [69] of the noise-contaminated videos of different JND models are measured, and the proposed model obtains optimal or suboptimal scores. The larger VMAF scores indicate that the subjective quality of the noise-contaminated sequences is closer to that of the original sequences. The results in Table 2 further verify the superiority of the proposed model in the VMAF metric scenario. Meanwhile, the average PSNR values of Bae 2017, Zeng 2019, Wang 2020, Xing 2021, Li 2022 and the proposed JND profile are 28.36 dB, 30.08 dB, 28.90 dB, 27.83 dB, 30.95 dB, and 27.03 dB, respectively. The average DMOS values of these are 12.49, 23.47, 24.08, 12.51, 19.59 and 9.42, respectively. The average VMAF values of these are 90.09, 87.71, 90.98, 97.30, 88.96 and 97.39, respectively. To visualize the data, Figure 15 displays the bar graphs of the average PSNR, VMAF and accompanying DMOS results for test sequences of various resolutions. Apparently, the noise-contaminated video generated by our model achieves the best perceptual quality (smallest DMOS score) and the largest distortion (lowest PSNR value) compared to the other four models. These experimental results validate the superiority of our JND profile in guiding noise injection.

**Figure 16:**
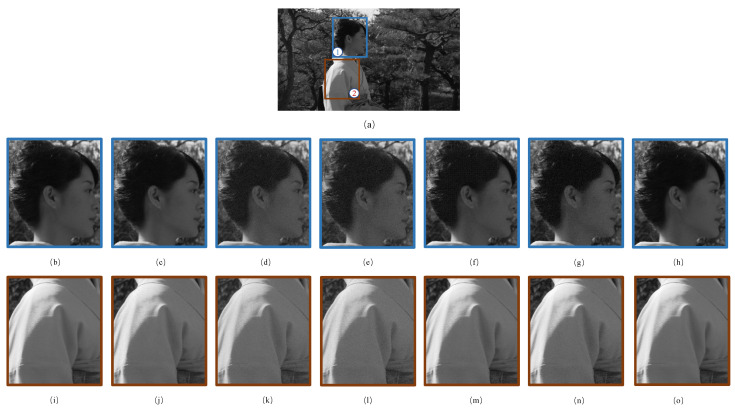
The 4th frame of the “Kimono1” sequence. (**a**) The original image; (**b**–**h**) are the enlarged images of the sub-region ①; (**i**–**o**) are the enlarged images of the sub-region ②. In turn, they are the original patch and the distorted versions processed by Bae 2017, Zeng 2019, Wang 2020, Xing 2021, Li 2022 and the proposed JND model, respectively. The PSNR values of the distorted versions are 27.53 dB, 28.17 dB, 27.76 dB, 27.10 dB, 27.98 dB and 26.69 dB, respectively.

In order to compare these five JND models more clearly, Figure 16 takes the 4th frame of the “Kimono1” sequence as an example. In general, HVS tends to pay more attention to the semantic targets [65]. In Figure 16a, the woman is prominent relative to the background. Therefore, a lower JND value should be applied in this region to inject less noise. We mainly focus on the head and body to allow for facilitate visual comparison. Apparently, there is considerable visible noise in Figure 16d–g, while the eyes and ears in Figure 16c are blurred. In contrast, Figure 16h is significantly clearer with the lowest PSNR value. As far as the body is concerned, the noise in Figure 16j–m is plainly apparent without exception, while in Figure 16o it is almost undetectable. As a consequence, the above objective and subjective evaluations validate that the proposed semantic attention-based weighting model is effective and excellent.

**Figure 17:**
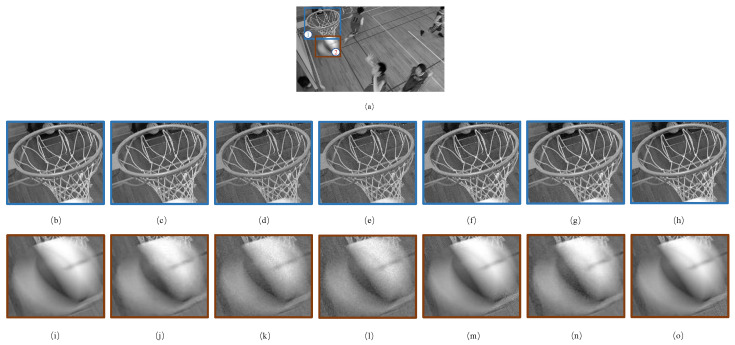
The 70th frame of the “Basketball Drill” sequence. (**a**) The original image; (**b**–**h**) are the enlarged images of the sub-region ①; (**i**–**o**) are the enlarged images of the sub-region ②. In turn, they are the original patch and the distorted versions processed by Bae 2017, Zeng 2019, Wang 2020, Xing 2021, Li 2022 and the proposed JND model, respectively. The PSNR values of the distorted versions are 27.24 dB, 32.12 dB, 30.63 dB, 26.16 dB, 33.42 dB and 25.42 dB, respectively.

As mentioned in the introduction, high-level semantic objects other than humans often appear in image and video frames. Frame 70 of the “Basketball Drill” sequence is depicted in Figure 17 as an illustration. Clearly, this is a scene depicting basketball training. Naturally, in this case, the basketball and the ball frame are obviously of interest in addition to the basketball players. With respect to the basketball frame, Figure 17c–g show significant distortion, while Figure 17h exhibits strong performance. For the basketball, Figure 17j–l,n depict considerable distortion, Figure 17m shows significant distortion at the lower right of the basketball, while the noise in Figure 17o is barely visible.

### 6.2. Ablation Experiment and Analysis

To verify the effectiveness of each module in this algorithm, this paper performed ablation experiments from three perception modules: object, context, and cross-object, and evaluated their impacts on the perception results.

The experimental results in Table 3 demonstrate that a combination of the three modules achieves optimal results, while the other combinations do not achieve the desired effect. In other words, when considering the influence of semantics on JND, one of the three aspects of object, context and cross-object features is indispensable. It is worth noting that since the context and cross-object modules are calculated based on object, combinations that do not contain object module are discarded. In practical applications, different kinds of videos focus on different scales of semantic features, and the combination of three modules achieves better generality.

## 7. Conclusions

A novel semantic-based JND model was proposed in this paper by thoroughly mining and characterizing the semantic feature parameters that affect attention. The interaction between semantic visual features and HVS was investigated from object, contextual and cross-object perspectives. This analysis included HVS responses induced by semantic sensitivity, objective area and central bias, as well as perceptual suppression brought about by contextual complexity and cross-object competition for attention. In conjunction with underlying attention in the spatial domain, perceptual attention to stimuli was measured with information-theoretic support and incorporated into a patch-level weighting factor. Using the semantic-based attentional weight, the JND model for the spatiotemporal transform domain was modified. The experimental results demonstrate the effectiveness of the proposed JND model with superior performance and stronger distortion-hiding ability compared to the state-of-the-art JND model.

Existing JND models only consider the effects of unimodal signals, while the study of cross-modal JND remains an open problem. However, there exists great incentive to transfer this issue from the laboratory to real-world adoption. In the future, we will further investigate multimodal asynchronous perception, seek a more unified approach to fusing multi-modal feature parameters, and obtain more accurate JND thresholds.

## Figures and Tables

**Table 1:** Performance comparison of different JND models.

Sequences	Bae 2017 [22]		Zeng 2019 [66]		Wang 2020 [12]		Xing 2021 [23]		Li 2022 [67]		Proposed	
	PSNR	DMOS	PSNR	DMOS	PSNR	DMOS	PSNR	DMOS	PSNR	DMOS	PSNR	DMOS
BasketballDrill	27.24	14.24	31.93	22.18	30.05	22.94	26.19	11.59	33.32	19.82	25.22	10.88
PartyScene	29.40	10.18	30.43	23.88	28.91	24.53	27.94	12.24	32.04	19.35	27.10	10.06
RaceHorses	31.27	9.59	30.58	22.76	29.57	23.47	29.50	10.47	31.48	21.06	29.96	8.71
FourPeople	26.86	14.41	29.06	24.41	28.38	24.94	25.90	14.88	29.86	19.29	25.30	11.94
Johnny	27.76	13.65	30.89	21.76	29.20	22.47	28.98	11.71	31.26	17.94	26.91	7.29
KristenAndSara	27.28	12.47	29.30	24.12	28.01	25.59	24.94	12.94	29.69	20.12	24.56	7.71
Kimono1	27.54	13.94	28.17	25.06	27.86	25.64	26.55	14.76	27.93	23.29	25.94	7.41
ParkScene	26.61	13.00	28.25	23.18	27.60	24.00	24.94	13.12	28.62	20.53	24.56	8.18
BasketballDrive	31.10	10.76	32.29	22.47	30.56	23.29	30.52	10.65	33.42	16.24	29.15	9.88
BQTerrace	28.54	10.53	29.89	23.65	28.90	23.88	29.73	12.71	31.88	18.29	28.54	8.12
**Average**	28.36	12.49	30.08	23.47	28.90	24.08	27.83	12.51	30.95	19.59	27.03	9.42

**Table 2:** Video multimethod assessment fusion scores.

Sequences	Bae 2017 [22]		Zeng 2019 [66]		Wang 2020 [12]		Xing 2021 [23]		Li 2022 [67]		Proposed	
	PSNR	VMAF	PSNR	VMAF	PSNR	VMAF	PSNR	VMAF	PSNR	VMAF	PSNR	VMAF
BasketballDrill	27.24	87.98	31.93	87.07	30.05	92.37	26.19	97.99	33.32	88.40	25.22	98.78
PartyScene	29.40	87.64	30.43	84.06	28.91	88.86	27.94	96.36	32.04	85.40	27.10	96.03
RaceHorses	31.27	93.36	30.58	90.40	29.57	98.65	29.50	99.91	31.48	91.61	29.96	99.92
FourPeople	26.86	89.66	29.06	87.48	28.38	87.63	25.90	93.07	29.86	88.54	25.30	93.10
Johnny	27.76	89.92	30.89	89.18	29.20	89.21	28.98	95.80	31.26	90.83	26.91	95.56
KristenAndSara	27.28	90.01	29.30	88.56	28.01	87.07	24.94	95.62	29.69	90.11	24.56	95.84
Kimono1	27.54	90.01	28.17	85.44	27.86	85.71	26.55	99.82	27.93	85.53	25.94	99.71
ParkScene	26.61	86.76	28.25	83.15	27.60	89.11	24.94	95.15	28.62	83.29	24.56	95.66
BasketballDrive	31.10	93.25	32.29	91.15	30.56	95.80	30.52	99.92	33.42	92.52	29.15	99.93
BQTerrace	28.54	92.08	29.89	90.56	28.90	95.40	29.73	99.40	31.88	93.40	28.54	99.35
**Average**	28.36	90.09	30.08	87.71	28.90	90.98	27.83	97.30	30.95	88.96	27.03	97.39

**Table 3:** Performance Comparison of Different Combinations of Modules.

Modules			PSNR		
**Object**	**Context**	**Cross-Object**	**832 × 480**	**1280 × 720**	**1920 × 1080**
✓			26.67	27.85	28.41
			29.95	32.07	27.91
			31.45	29.51	31.09
					29.80
✓	✓		25.66	26.07	26.53
			28.30	29.26	25.02
			30.32	27.48	30.19
					29.12
✓		✓	25.56	26.10	26.32
			28.22	28.78	25.67
			30.28	28.24	30.70
					28.97
✓	✓	✓	25.22	25.30	25.94
			27.10	26.91	24.56
			29.96	24.56	29.15
					28.54
